# Study on the Influence of Nanosilica Sol on the Hydration Process of Different Kinds of Cement and Mortar Properties

**DOI:** 10.3390/ma14133653

**Published:** 2021-06-30

**Authors:** Haibao Liu, Qiuyi Li, Dunlei Su, Gongbing Yue, Liang Wang

**Affiliations:** 1 School of Civil Engineering, Qingdao University of Technology, Qingdao 266033, China; lhbhxw@163.com; 2 School of Architectural Engineering, Qingdao Agricultural University, Qingdao 266109, China; yuegongbing@163.com (G.Y.); jiangongwl_2019@163.com (L.W.); 3 School of Civil Engineering, Shandong Jiaotong University, Jinan 250357, China

**Keywords:** nanosilica sol, hydration heat analysis, SEM observation, mechanical strength, chloride penetration resistance

## Abstract

Compared with nanosilica collected in a gaseous state, nanosilica sol has great economic value and application significance for improving the performance of concrete and mortar. In this study, the influence of nanosilica sol on the hydration process of different kinds of cement is studied by means of hydration heat analysis, X-ray diffraction analysis (XRD) and other methods, and the properties of mortar such as setting time, mechanical properties and porosity are also studied to characterize the influence of nanosilica sol on the macroscopic properties of mortar. The experimental results show that nanosilica sol can accelerate the hydration rate of two kinds of cement and promote the hydration reaction degree of cement, and this promotion effect increases with the increase in nanosilica sol content. At the same time, nanosilica sol can significantly shorten the setting time of the two kinds of cement, and it is more obvious with the increase in content. Excessive content of nanosilica sol will adversely affect the permeability resistance of mortar. It may be caused by the weak interval formed by nanosilica particle clusters in the mortar matrix, which can be supported by the mortar pore structure distribution test. At the same time, the influence of nanosilica sol on the hydration of the two kinds of cement is different, and the compressive strength of HBSAC cement mortar increases first and then decreases after adding nanosilica sol; However, the compressive strength of P·O 42.5 cement mortar increases gradually after adding nanometer silica sol. This shows that nanosilica sol does not effectively promote the hydration of β-C2S in high belite sulfoaluminate cement (HBSAC) mortar. Based on the above experimental results, it can be concluded that when the content of nanosilica sol is about 1%, it has the best promotion effect on the hydration of the two kinds of cement and the performance of mortar.

## 1. Introduction

Mineral admixtures have been widely used in concrete for a long time. They can effectively improve the microstructure of concrete and then further affect the mechanical properties and durability of concrete [1,2,3]. Nanomaterials are considered the most potential materials of the 21st century. At the same time, nanomaterials have also been widely used in concrete. Relevant research shows that compared with commonly used mineral admixtures, nanosilica has a finer particle size, higher pozzolanic activity and a seeding effect [4,5,6,7,8,9,10,11,12,13,14], so the application of nanosilica in concrete has better prospects than other mineral admixtures. Although there have been sufficient studies showing that a lot of benefits can be introduced into cement-based materials by mixing nanosilica, there are still many problems in practical application. When nanosilica is added to cement-based materials, the agglomeration of nanosilica particles will occur. The reason for this phenomenon may be that the production process of nanosilica makes the surface of the particles have huge surface energy, coupled with the high pH generated after the hydration and dissolution of cement and the complex environment containing multiple ions. The nanosilica particles are agglomerated, and this agglomeration phenomenon will damage the performance of concrete and mortar [15,16,17,18]. Ghafari et al. [19] showed that the incorrect dispersion of nanosilica will increase the amount of nanosilica in cement-based materials. In addition, nanosilica clusters become harmful pores in cement-based materials after hydration and hardening. The results show that [20] compared with C–S–H gel formed by the hydration of cement, C–S–H gel formed by the reaction of nanosilica clusters and calcium hydroxide (CH) has lower binding performance. At the same time, [21,22] the decrease in mechanical properties of cement-based materials is also due to the poor dispersion of NS in cement-based materials. In addition, after NS particles were aggregated, the number of seeds decreased significantly [23].

Compared with the powdered nanosilica, nanosilica sol has special advantages in particle dispersion. Nanosilica sol can uniformly disperse nanosilica particles in aqueous solution by the hydrolysis of silicon powder, and at the same time, stabilizers such as ammonium chloride are added to the solution so that nanosilica can mostly exist in submicron particle aggregates [24,25] in the solution, thus better exerting crystal nucleation and filling functions. At the same time, many researchers have carried out a series of studies on the influence of nanosilica sol on the properties of cement-based materials. Zhang [26] showed that the particle size of colloidal nanosilica has an influence on the performance of fresh mortar. The results show that when the particle size is 60 nm, nanosilica has the best effect on improving the performance of concrete. Ghasemi et al. [27] showed that colloidal nano-SiO_2_, as a substitute material for silica fume, can improve the permeability resistance and the compressive strength of concrete. In addition, Kong [28,29] showed that colloidal silica sol has a good effect on cement hydration and concrete microstructure regulation and has more advantages than nanosilica powder.

High-belite sulphoaluminate cement is a kind of green cement [30,31] that consumes less energy and emits less CO_2_ in the preparation process. At the same time, properties such as quick setting and hardening, low hydration heat and excellent permeability resistance make high-belite sulphoaluminate cement widely useful in many fields, such as road rapid repair. However, high-belite sulphoaluminate cement still has some defects, such as the slow hydration reaction speed of C_2_S, which leads to the insignificant long-term strength growth of cement. Whether the addition of nanosilica sol can improve this shortcoming is a subject worthy of our attention. At the same time, P·O 42.5 cement, as the largest cement variety, has been widely used in construction. By adding nanosilica sol, the different influence laws on the hydration process of the two kinds of cement can be obtained, which has a positive significance for exploring the application of nanosilica sol in different cement-based materials.

Therefore, in this study, hydration heat analysis, X-ray diffraction analysis (XRD), thermogravimetric analysis (TGA) and scanning electron microscope(SEM) observation were mainly conducted to analyze the hydration process of different kinds of cement and the product mineral composition. The influence of nanosilica sol on the macro–micro properties of cement-based materials is revealed.

## 2. Experiment

### 2.1. Raw Materials

In this study, a type of nanosilica sol was obtained from Dezhou Keying New Materials Co., Ltd. (Dezhou, China). High-belite sulphoaluminate cement (HBSAC) was provided by Tangshan Polar Bear Special Cement Co., Ltd. (Tangshan, China). Ordinary Portland cement (P·O 42.5 grade) was by Qingdao Shanshui Cement Co., Ltd. (Qingdao, China). The water-reducing agent from Qingdao New Building Materials Co., Ltd. (Qingdao, China) was used. The main chemical composition of the raw materials by XRF analysis is shown in Table 1. The nanosilica sol was produced by the silica fume hydrolysis method, and the sizes of the nanosilica particles were between 15 and 30 nm.

### 2.2. Design of Experiment

**Figure 1:**
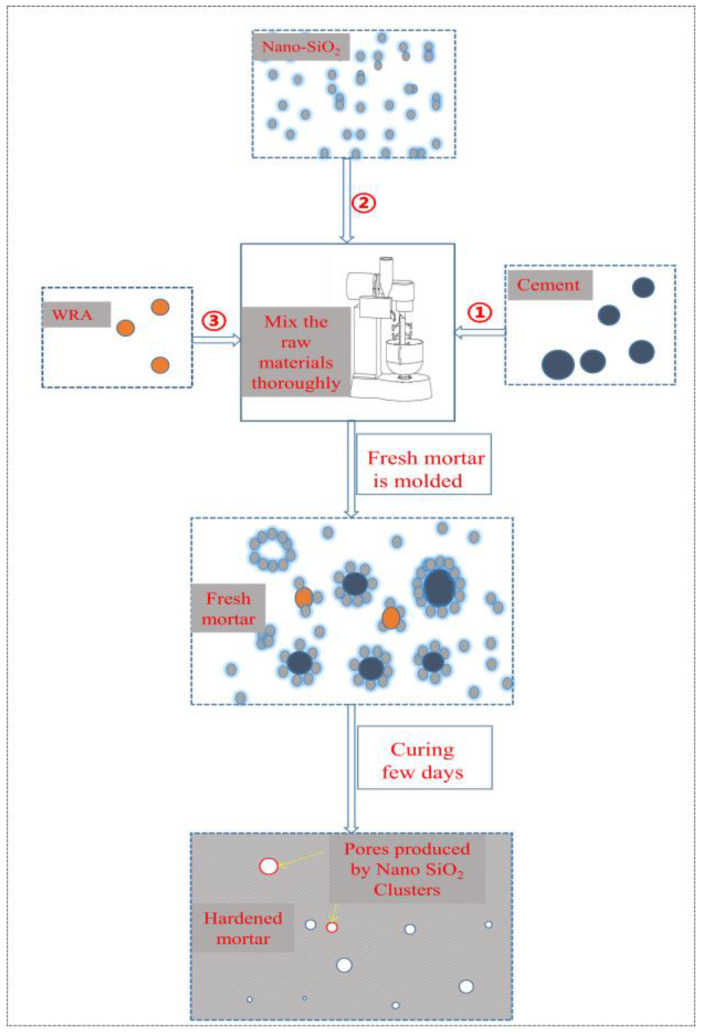
Experimental procedure diagram.

The addition mixing water and water-reducing agent for mortar is based on the premise of ensuring the same fluidity of each group of test mortar. Referring to the test method of “Sulphoaluminate Cement” (GB 20472-2006, China), the fluidity of each group of test mortar was controlled at 170 ± 10 mm, and the fluidity of the mortar was adjusted by adding the water-reducing agent. The specific experimental mix proportion design is shown in Table 2. After the raw materials were fully stirred and mixed, the freshly mixed mortar was molded. Then, the demolded pastes were cured at 20 ± 1 °C and over 95% relative humidity for 3 d, 7 d and 28 d. Because the adding order of raw materials of mortar may affect the final form and application effect of nanosilica sol in mortar, in the mortar experiment, each raw material was added in sequence according to the sequence shown in Figure 1. In the mortar mixing test, the adding order of each raw material was cement, nanosilica sol, water and water-reducing agent.

### 2.3. Experimental Methods

#### 2.3.1. SEM Observation

The influence of nanosilica sol on the microstructure of the different kinds of cement was observed through SEM observation analysis. After the pastes were cured at 20 ± 1 °C and over 95% relative humidity, they could be compared and analyzed through the heat release curve of hydration heat, and the influence of nanosilica sol on the hydration process of cement could be distinguished.

#### 2.3.2. XRD Analysis

The mineral composition of cement hydration was detected by using an X-ray diffractometer (D9 Advanced Type, Bruker Co., Karlsruhe, Germany) with relative humidity for 28 days. Then, the testing sample was washed with ethanol, vacuum-dried at 105 ± 5 °C for 10 h and detected by SEM (JSM-7500F type, JEOL Co., Ltd., Tokyo, Japan). The composition and distribution of cement hydration products in specific areas could be analyzed by SEM observation, which provided a more objective basis for the experimental results.

#### 2.3.3. Hydration Heat Analysis

The hydration heat test was carried out by using a TAM Air eight-channel microcalorimeter (TAM Air, TA instrument Co. Newcastle, DE, USA), and the operating temperature range of TAM Air was 5–90 °C. The hydration temperature was measured by the adiabatic method. A calorimeter was used to evaluate the effect of nanosilica on the hydration heat of cement paste. Testing samples were prepared at a constant W/B ratio of 0.4, requiring a total of 4 g of cement samples and 1.6 g of water. A constant temperature tank used circulating air as the medium, and temperature stability was ensured by an automatic adjustment system. The recording time of the hydration heat of cement samples lasted for 72 h. The influence of different content of nano-silica sol on the hydration process of cement was studied. The working conditions of the machine were: copper target, 45 kV voltage, 50 mA current, 5°–60° 2-theta scanning range, 0.02 step width and 0.05 s residence time. A cement paste test specimen with a size of 20 × 20 × 20 mm^3^ was made, and when the test specimen was cured to a specified curing age, the test specimen was ground into a powder to make a sample. XRD analysis can judge the hydration degree of cement and whether the corresponding reaction occurs from the perspective of hydration products so that the experimental conclusion can be objectively supported.

#### 2.3.4. MIP Analysis

The type of equipment machine used to test the pore distribution of mortar was an autopore IV 9500 (Micromeritics Co., Atlanta, GA, USA), and the pore size test range was 0.003–1000 m. The size of the test specimen was 1 × 1 × 1 mm^3^. Afterwards, the pastes were cured at 20 ± 1 °C and over 95% relative humidity for 28 days, washed with ethanol and vacuum-dried at 105 ± 5 °C for the target age.

#### 2.3.5. Thermal Analysis

A comprehensive thermal analyzer (SDT Q600, TA Instruments Co., New Castle, DE, USA) was used for differential thermal analysis of the hydration product. The temperature ranged from 20 °C to 800 °C, the heating rate was 25 °C/min and the flow rate was N2. The hydration product sample was the same as the sample used for XRD analysis.

#### 2.3.6. Setting Time and Mechanical Properties

According to the setting time determination method of ordinary Portland cement and high-belite sulphoaluminate cement, referring to the “Test Method for Water Requirement, Setting Time and Stability of Portland Cement Standard Consistency” (GB/T 1346-2011, China), the setting time determination method of ordinary Portland cement and high-belite sulphoaluminate cement was used. The mechanical test method of mortar strength refers to the specification “Sulphoaluminate Cement” (GB 20472-2006, China). The size of the test specimen was 40 × 40 × 160 mm^3^, in which the water demand for mortar mixing was controlled by the fluidity of mortar, and the fluidity was 170 mm. After the mortar test blocks were prepared, all the test specimens were naturally cured for 1 day and then demolded and solidified. Subsequently, it was placed in an environment with a temperature of 20 ± 1 °C and relative humidity of no less than 95% to be cured until target ages. Three samples were used for the setting time test of each group of mortar, and three samples were used for the compressive strength test of each group of mortar at each test age.

#### 2.3.7. Chloride Penetration Resistance

The rapid chloride ion migration coefficient method (or D_RCM_ method) of “Standard for Test Methods of Long-term Performance and Durability of Ordinary Concrete” (GB/T 50082-2009) as used to evaluate the chloride ion penetration resistance of concrete. The unsteady chloride ion migration coefficient of mortar is calculated by the following formula:

(1) $$D_{RCM}=\frac{0.0239\left(273+T\right)L}{\left(U-2\right)t}\left(X_{d}-0.0238\sqrt{\frac{\left(273+T\right)LX_{d}}{U-2}}\right)$$

In the formula, *U* represents the absolute value (V) of the voltage used in the test; *T* represents the average value (°C) of the initial temperature and the end temperature of the anode solution; *L* represents the thickness (mm) of the test piece; *X_d_* represents the chloride ion penetration depth; *t* represents the duration of the test (h).

## 3. Results and Discussions

### 3.1. Hydration Heat Analysis

**Figure 2:**
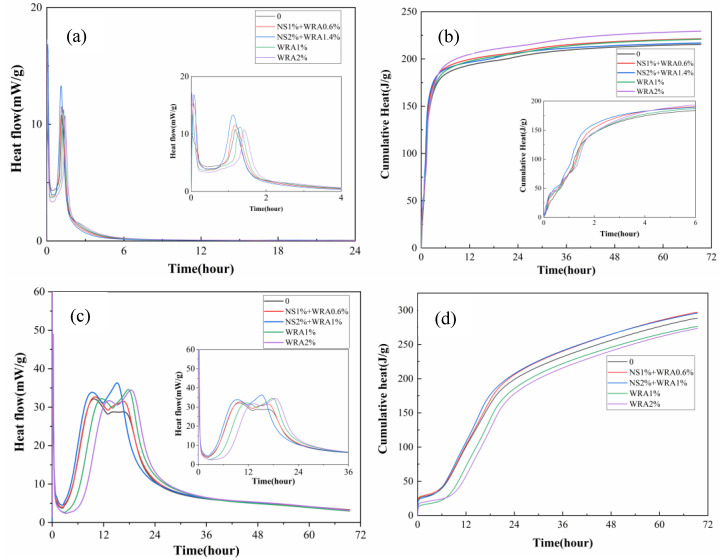
Hydration heat curves of the series cement with nanosilica sol: (**a**) heat flow curve of HBSAC cement; (**b**) cumulative heat curve of HBSAC cement; (**c**) heat flow curve of P·O 42.5 cement; (**d**) cumulative heat curve of P·O 42.5 cement.

The influence of nanosilica sol with different dosages on the hydration heat release rate of cement is shown in Figure 2a,c. It can be clearly seen from Figure 2a that HBSAC cement has two obvious exothermic peaks.

From Figure 2, it can be concluded that the hydration speed and hydration degree of the two kinds of cement are improved to different degrees by adding nanosilica sol. For HBSAC cement, the first exothermic peak is mainly caused by the heat of the hydration heat and dissolution, and the second exothermic peak is mainly caused by hydration in the cement. The second exothermic peak time of HBSAC cement with 0/1% and 2% nanosilica sol is 1.3 h, 1.23 h and 1.1 h, respectively. Similarly, compared with the control group, when the content of nanosilica sol is 1%, the appearance time of the second and third exothermic peaks of P·O 42.5 can be advanced by 0.2 h and 0.3 h, respectively. Similarly, when the content of nanosilica sol is 2%, the second and third exothermic peaks of P·O 42.5 cement can be advanced by 1 h and 2.5 h, respectively. Therefore, the experimental results show that nanosilica can promote the hydration rate of the two kinds of cement, and this phenomenon gradually becomes obvious with the increase in nanosilica content. This phenomenon may be due to the “seeding effect” of nanosilica. Nanosilica sols are fully dispersed in fresh mortar, and those particles can provide seeding sites when cement particles gradually hydrate, which will accelerate cement hydration. At the same time, in order to eliminate the influence of the water-reducing agent on cement hydration, an experimental group with the water-reducing agent alone was set up. The test results showed that this water-reducing agent has a retarding effect on the hydration of both cements, and this phenomenon becomes obvious with the increase in water-reducing agent content.

**Figure 3:**
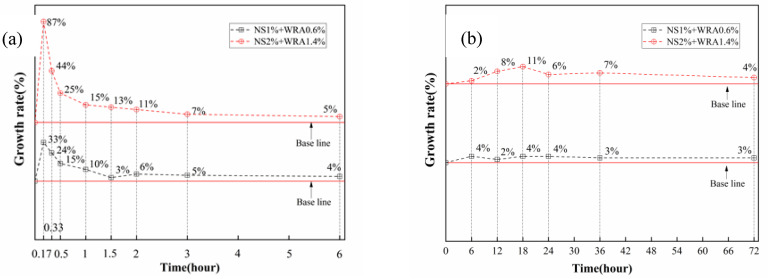
Growth rate of cumulative hydration heat: (**a**) HBSAC cement; (**b**) P·O 42.5 cement.

The cumulative heat of hydration of HBSAC cement and P·O 42.5 cement is shown in Figure 2b,d, respectively, and Figure 3 shows the growth rate of the cumulative heat of HBSAC cement and P·O 42.5 cement by adding nanosilica sol.

The promotion degree of adding nanosilica to cement hydration can be quantitatively obtained. As can be seen from Figure 3a, the hydration promotion degree of nanosilica sol to HBSAC cement and P·O 42.5 cement reached a peak at 20 min and 18 h, respectively, and then the promotion degree gradually decreased with the progress of the hydration reaction. Similarly, it is observed from the figure that the degree of promotion for the hydration of the two kinds of cement becomes more significant as the content of nanosilica sol increases.

### 3.2. Hydration Products Analysis

#### 3.2.1. XRD Analysis

The hydration reaction of P·O 42.5 cement consists of the following reactions:

(2) $$C_{3}S+3H\to C-S-H(\mathrm{gel})+2\mathrm{CH}$$

(3) $$C_{2}S+2H\to C-S-H\left(\mathrm{gel}\right)+\mathrm{CH}$$

**Figure 4:**
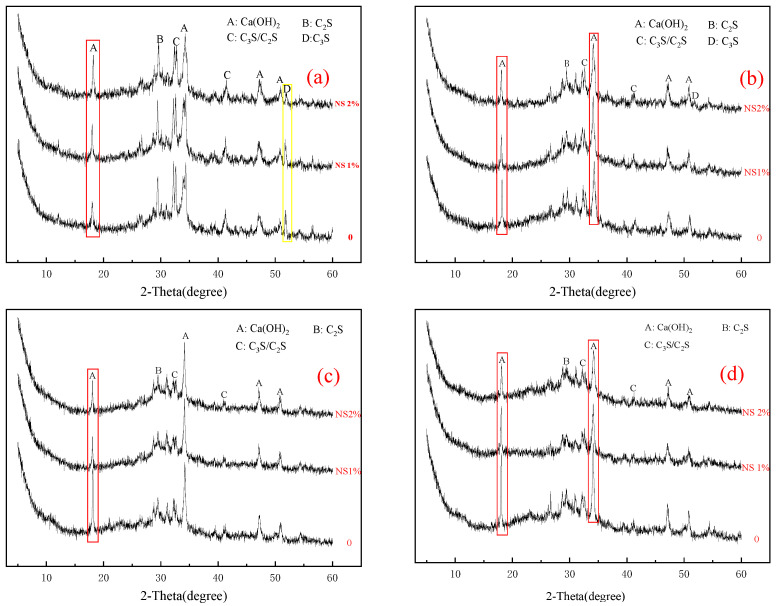
X-ray diffraction (XRD) patterns of the P·O 42.5 cement with different contents of nanosilica sol: (**a**) 1 day; (**b**) 3 days; (**c**) 7 days; (**d**) 28 days.

Figure 4 shows that compared with the control group, the peak heights of cement mineral components C_3_S and β-C_2_S will gradually decrease with the increase in silica content, which means that nanosilica can promote the hydration of P·O 42.5 cement. At the same time, the peak height of Ca(OH)_2_, a hydration product of cement, gradually decreases with the increase in silica content. This phenomenon may be caused by the “secondary hydration reaction” [25,26,32,33] between some Ca(OH)_2_ and nanosilica. The hydrated calcium silicate gel (C–S–H) generated by this reaction will fully fill the pores in the cement paste. This phenomenon has a positive effect on the performance of mortar or concrete.

The HBSAC cement hydration reaction consists of the following reactions:

(4) $$C_{4}A_{3}\bar{S}+2C\;\bar{S}+38H\to C_{3}A\cdot 3C\;\bar{S}\cdot H_{32}+2{\mathrm{AH}}_{3}$$

(5) $$C_{2}S+2H\to C-S-H\left(\mathrm{gel}\right)+\mathrm{CH}$$

**Figure 5:**
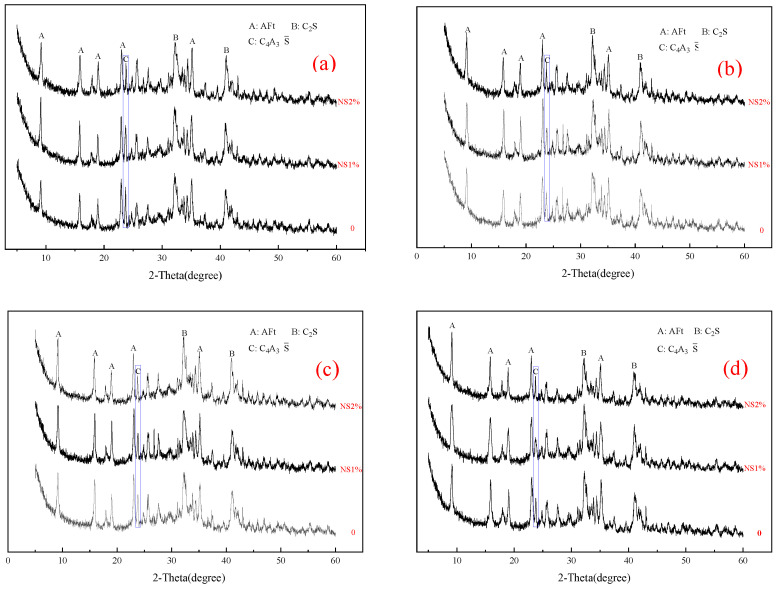
X-ray diffraction (XRD) patterns of the HBSAC cement with different contents of nanosilica sol: (**a**) 3 h; (**b**) 6 h; (**c**) 1 day; (**d**) 28 days.

The hydration rate of anhydrous calcium sulphoaluminate ($C_{4}A_{3}\bar{S}$) is shown in Equation (4), and its hydration rate is extremely fast, which can make the 1 d strength of high-belite sulphoaluminate cement reach more than 85% of the final strength [31,34]. As can be seen from Figure 5, the content of hydrated calcium sulphoaluminate increases with the increase in nanosilica content at the same cement age, which also indicates that nanosilica can further promote the hydration process of anhydrous calcium sulphoaluminate ($C_{4}A_{3}\bar{S}$). However, the content of C_2_S does not decrease obviously with the increase in the content of nanosilica sol, which indicates that nanosilica sol has a limited effect on the hydration of C_2_S.

#### 3.2.2. TGA Analysis

**Figure 6:**
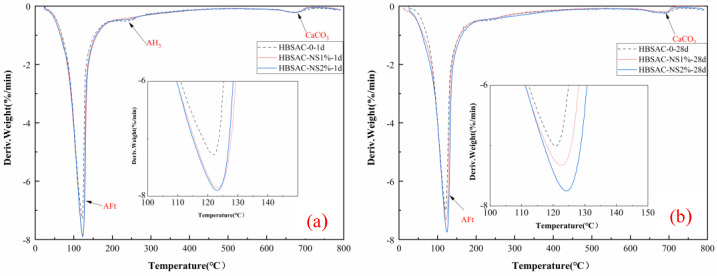

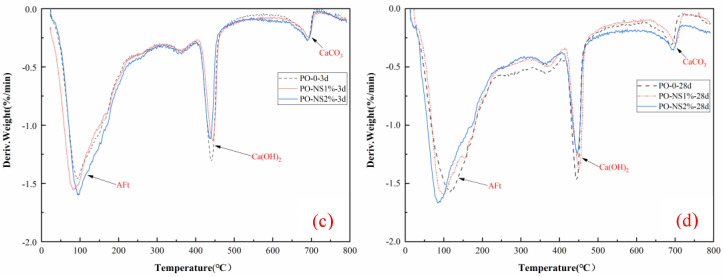
DTG curves of the series cement hydration products at different curing ages: (**a**) HBSAC cement at 1 day; (**b**) HBSAC cement at 28 days; (**c**) P·O 42.5 cement at 1 day; (**d**) P·O 42.5 cement at 28 days.

Figure 6 shows the thermogravimetric loss curves of the cement hydration products at different curing ages.

The dehydration decomposition temperature of hydrated calcium sulphoaluminate (AFt), a type of HBSAC hydration product, is generally 100–150 °C. The dehydration decomposition temperature of calcium hydroxide (Ca(OH)_2_) is generally 350–550 °C [35,36]. Calcium hydroxide (Ca(OH)_2_) in the HBSAC cement hydration product is produced by the reaction shown in Equation (5). The experimental results show that there is almost no dehydration decomposition of calcium hydroxide (Ca(OH)_2_) except AFt. However, the hydration degree of C_2_S did not change obviously with the addition of nanosilica sol. At the same time, it also means that the early performance of HBSAC cement mortar almost comes from the hydration of anhydrous calcium sulphoaluminate. Moreover, with the increase in nanosilica sol content, the mass loss rate of hydrated calcium sulphoaluminate (AFt) increases, which also means that nanosilica sol can promote the hydration reaction of $C_{4}A_{3}\bar{S}$. This phenomenon is basically consistent with the XRD experimental results.

The dehydration decomposition temperature of calcium silicate hydrate (C–S–H), one of the hydration products of P·O 42.5 cement, is generally 100–400 °C. From Figure 6c,d, it can be concluded that the content of calcium hydroxide (Ca(OH)_2_) decreases with the increase in nanosilica content, which may be caused by the “secondary hydration reaction” between nanosilica and calcium hydroxide (Ca(OH)_2_). This phenomenon is consistent with the above XRD analysis conclusion. There is a dehydration decomposition peak in the range of 700–800 °C, and the product may be calcite, which is probably caused by the carbonization of calcium hydroxide (Ca(OH)_2_) by protective gas during the test [37].

### 3.3. SEM Micromorphology

**Figure 7:**
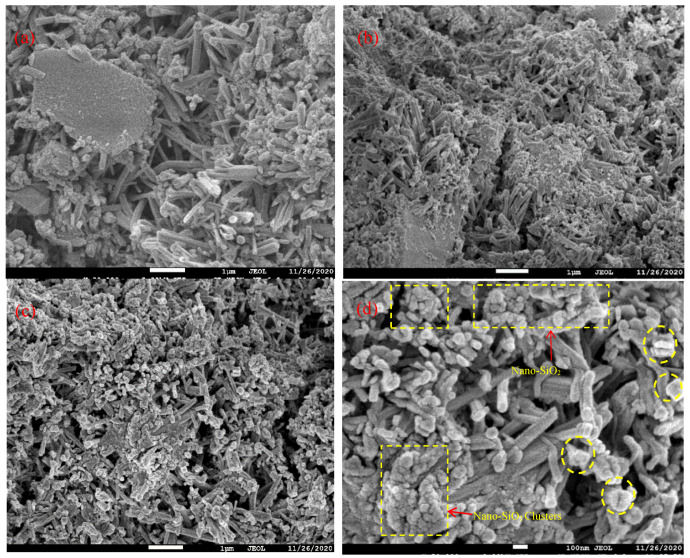
SEM micromorphology of the HBSAC cement hydration products with different contents of nanosilica sol: (**a**) nanosilica sol content 2% at 1 day; (**b**) nanosilica sol content 0 at 1 day; (**c**) nanosilica sol content 2% at 28 days; (**d**) nanosilica sol content 2% at 28 days.

Figure 7 shows the SEM micromorphology of HBSAC cement to which nanosilica sol is added. The hydration products of high-belite sulphoaluminate cement HBSAC are mainly rod-shaped ettringite (AFt).

The microscopic morphology of the ettringite phase, the main hydration product of HBSAC cement, is needle-rod shaped. Compared with the control group, it can be seen that the acicular ettringite in HBSAC cement added with nanosilica sol becomes coarse. Ettringite overlaps alternately to form a skeleton, which is filled with gel phases such as aluminum glue to fill gaps and cementation, thus making the hardened cement paste denser [38,39]. The reason for this phenomenon may be that the crystal nucleus of nanomaterials plays a role, which is also strong proof that nanosilica promotes cement hydration.

From Figure 7c,d, it can be seen that the nanosilica is not uniformly distributed in the cement paste. Nanosilica has great surface energy, and it will agglomerate when mixed with cement particles. Some of the nanosilica particles are adsorbed around the cement particles, and only some of the nanosilica particles are evenly dispersed in the paste. The distribution of silica in cement paste is shown in Figure 1. These agglomerates may form weak areas such as pores inside the paste, which can become an incentive to weaken the performance of concrete and mortar [40].

**Figure 8:**
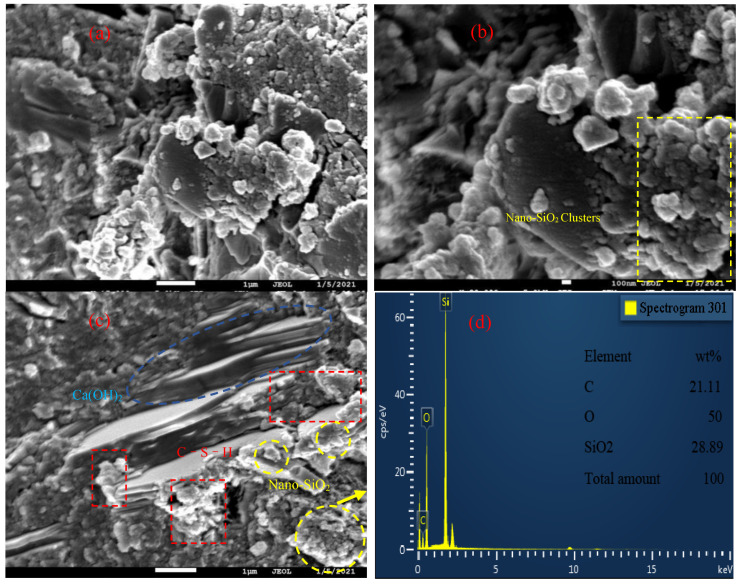
SEM micromorphology of the P·O 42.5 cement hydration products at curing ages of 28 days: (**a**–**c**) “secondary hydration reaction” between nanosilica particles and calcium hydroxide; (**d**) EDS image of nanosilica sol clusters (the test points are taken from (**c**)).

Figure 8 shows the SEM micromorphology of P·O 42.5 cement paste with nanosilica sol. From Figure 8a,b, it can be seen that the mechanism of nanosilica sol in P·O 42.5 cement is different from that in HBSAC. Similarly, the nanosilica sol will be adsorbed around the cement particles when the mortar is mixed and act as a nucleus site when the cement particles are hydrated, promoting the hydration of the cement. At the same time, the nanosilica dispersed around the cement hydration product Ca(OH)_2_ will react to form calcium silicate hydrate sol (C–S–H). This reaction is undoubtedly beneficial to the performance of mortar and concrete. Hydrated calcium silicate sol (C–S–H) can fill the pores inside the paste, making the paste denser.

### 3.4. Workability and Mechanical Strength

#### 3.4.1. Workability

**Figure 9:**
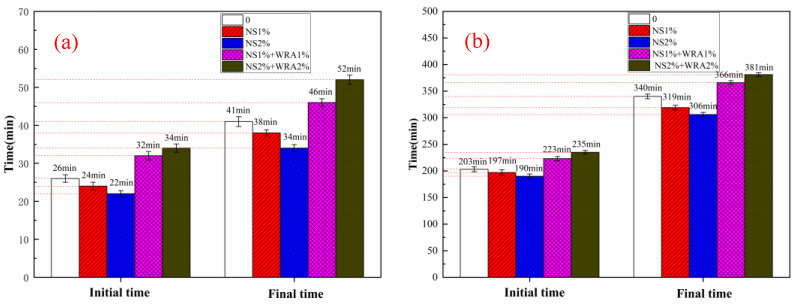
Setting time of different cements: (**a**) HBSAC cement; (**b**) P·O 42.5 cement.

Figure 9 shows the setting time of a cement paste with different contents of nanosilica sol. When the content of nanosilica sol is 1% and 2%, the initial setting time of P·O 42.5 cement is shortened by 2 min and 4 min, respectively, and the final setting time is shortened by 3 min and 7 min, respectively. Similarly, compared with the control group, when the content of nanosilica sol is 1% and 2%, the initial setting time of HBSAC cement is shortened by 6 min and 13 min, respectively, and the final setting time is shortened by 21 min and 34 min, respectively. The promoting effect of nanosilica sol on cement hydration can also be reflected in cement setting time. The experimental results show that the addition of nanosilica sol reduces the initial setting time and final setting time of the two kinds of cement to a certain extent, and this phenomenon becomes more obvious with the increase in nanosilica content. This change trend is consistent with the results of hydration heat analysis, which is strong proof that nanosilica sol can promote cement hydration.

#### 3.4.2. Mechanical Strength

The strength test method of cement mortar refers to GB/T 17671-2020, “Test Method for Strength of Cement Mortar”. In the experiment, the experimental group without nanosilica was set as the control group.

**Figure 10:**
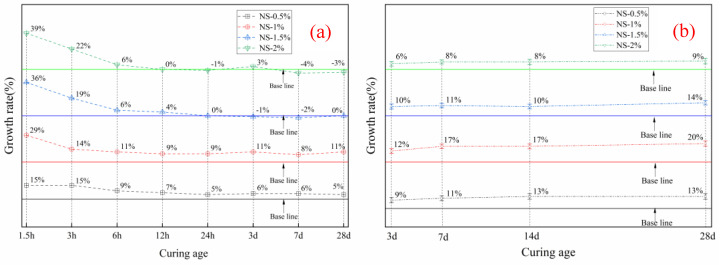
Growth rate of the cement mortar compressive strength: (**a**) HBSAC cement; (**b**) P·O 42.5 cement.

The test results show that the influence of nanosilica sol on the strength of HBSAC cement and P·O 42.5 cement is different. Firstly, the influence of nanosilica sol on the strength of HBSAC cement is related to its curing age. In the early hydration stage of HBSAC cement, adding nanometer silica sol can effectively improve the strength of cement mortar. When the curing age was more than 3 h, this promotion effect began to decrease gradually Secondly, the content of nanosilica sol has the best content for the strength of cement mortar, which is about 1%. The main hydration reaction of HBSAC cement is shown in Equation (2), and the hydration reaction is carried out rapidly and can be completed quickly within 3 h. From the XRD analysis of the hydration product, it can be concluded that there is almost no Ca(OH)_2_ in the hydration product, which means that the pozzolanic activity of nanosilica cannot be exerted during the hydration reaction of HBSAC cement paste. As shown in Figure 10 and Figure 8c, nanosilica clusters form pores in the paste, and the pores will increase with the increase in nanosilica sol content. These pores form a deteriorated interface, which will adversely affect the strength of mortar.

The promotion effect of nanosilica sol on the strength of P·O 42.5 cement gradually increases with the increase in curing age. Secondly, with the increase in the content of nanosilica sol, the promotion effect shows an increasing trend at first and then a decreasing trend, and the best content is about 1%. As shown in Figure 8c, a large amount of Ca(OH)_2_ generated by the hydration reaction of ordinary Portland cement will rapidly undergo a hydration reaction with nanosilica adsorbed around, and the generated hydrated calcium silicate gel (C–S–H) can effectively fill the pores inside the paste, making the paste denser, thus effectively promoting the strength increase of mortar.

### 3.5. Porosity

**Figure 11:**
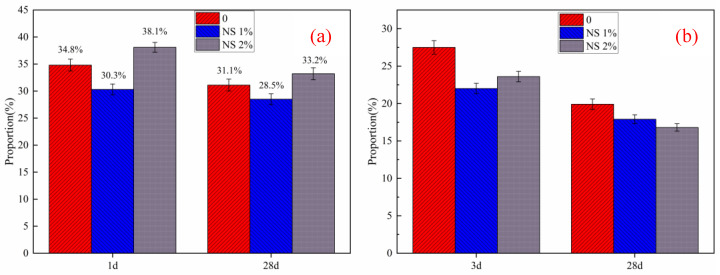
Porosity of cement mortar: (**a**) HBSAC cement; (**b**) P·O 42.5 cement.

Figure 11 shows the porosity of cement paste with different amounts of nanosilica sol. The test results show that the proper content of nanosilica sol can effectively reduce the porosity of mortar, but too-high content will lead to an increase in porosity, which is applicable to both HBSAC cement and P·O 42.5 cement. The research results of Quercia [41] show that a part of nanosilica particles mixed in fresh mortar will be adsorbed around cement particles and water-reducing agent particles. After the cement paste is hardened, nanosilica particles can effectively fill the pores in the cement paste, which is exactly the embodiment of nanosilica “microaggregate effect”. The data show that the best content of nanosilica sol is about 1%. However, nanosilica usually exists in the form of clusters, which will leave pores in the cement paste after hardening. This phenomenon can also explain why a large amount of nanosilica sol will increase the porosity of the cement paste.

**Figure 12:**
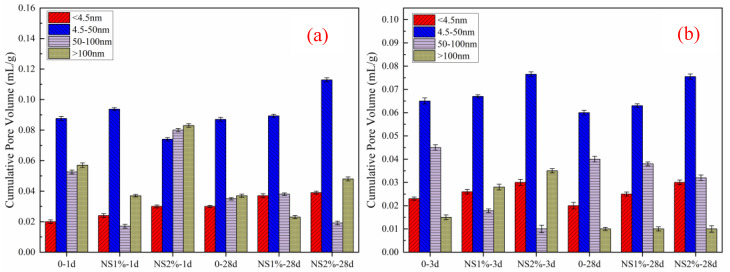
Pore size distribution of cement paste: (**a**) HBSAC cement; (**b**) P·O 42.5 cement.

Figure 12 shows the pore size distribution of cement paste. The research results of Metha [42] show that the pores in the cement paste can be divided into four categories according to the pore diameter: less than 4.5 mm, 4.5–50 mm, 50–100 mm and greater than 100 mm, of which only the pores greater than 100 mm are called harmful pores. Too many harmful holes will adversely affect the strength and permeability of mortar. As shown in Figure 12, doping nanometer silica sol can effectively optimize the pore size distribution inside the mortar paste, but too high a doping amount will lead to an increase in harmful pores, and the optimal doping amount of nanometer silica is about 1%. With the increase in curing age, nanosilica can optimize the pore structure in paste, which makes the number of harmful pores drop sharply. In addition, there is an interesting phenomenon when adding the same amount of nanosilica, the harmful pores of HBSAC cement are more than those of P·O 42.5 cement. This phenomenon may be caused by the reaction between nanosilica clusters and calcium hydroxide to reduce the number of harmful pores in cement mortar, while HBSAC cement does not have this reaction characteristic.

### 3.6. Chloride Penetration Resistance

**Figure 13:**
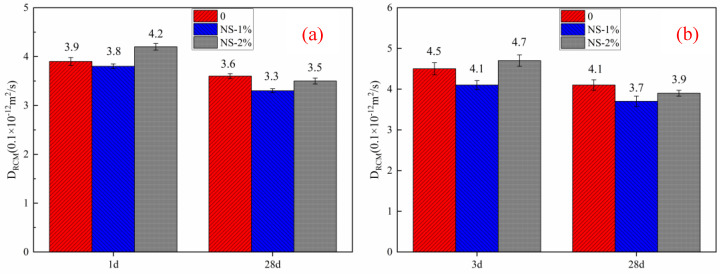
Chloride penetration resistance of cement mortar: (**a**) HBSAC cement; (**b**) P·O 42.5 cement.

Figure 13 shows the chloride penetration resistance of mortar with different contents of nanosilica sol. Chloride penetration resistance of mortar is one of the most important performance indexes of mortar and concrete because it directly determines whether the steel bars in concrete can be effectively protected. The test results show that at the early age of mortar, the content of nanosilica is about 1%, which contributes the most to the chloride penetration resistance of mortar. However, when the content of nanosilica sol reaches 2%, it will adversely affect the chloride penetration resistance of mortar, which is applicable to both HBSAC cement and P·O 42.5 cement. This change trend is consistent with the change law of the porosity of cement mortar. This phenomenon may be due to the filling effect and the secondary hydration reaction of nanosilica, which leads to a reduction in harmful pores in cement paste and makes the paste denser.

## 4. Conclusions

In this study, hydration heat analysis, SEM observation and XRD analysis were used to study the changes of nanosilica sol on the hydration process and micromorphology of HBSAC cement and P·O 42.5 cement, mechanical strength, porosity, chloride penetration resistance and other performance indicators of mortar, and the following results and conclusions are obtained.

1. Adding nanometer silica sol can not only accelerate the hydration speed of cement but also promote the hydration degree of cement, and this phenomenon is applicable to both kinds of cement. The greater the content of nanosilica sol, the more obvious the promotion effect. The promotion effect of nanosilica sol on the cumulative heat of hydration of HBSAC cement reached the peak at about 20 min, and that of P·O 42.5 cement reached the peak at about 20 min.
2. From the analysis of cement hydration products, it is concluded that for high-belite sulphoaluminate cement (HBSAC), nanosilica sol can effectively promote the hydration of anhydrous calcium sulphoaluminate, but the hydration promotion effect on β-C_2_S is weak. For ordinary Portland cement, nanosilica sol can effectively promote the hydration process of C_3_S, and the content of calcium hydroxide (CH) in hydration products decreases with the increase in nanosilica sol content, which may be due to the “secondary hydration reaction” between calcium hydroxide (CH) and nanosilica.
3. SEM observation shows that nanosilica sol can accelerate the hydration rate of HBSAC cement, and the hydration products grow faster. At the same time, nanosilica sol can react with calcium hydroxide, which the P·O 42.5 cement hydration reaction produces, to form hydrated calcium silicate gel. However, the agglomeration of nanosilica particles in the cement paste can also be observed, which may adversely affect the pore structure and permeability resistance of the mortar.
4. MIP analysis and chloride penetration resistance showed that nanosilica sol clusters have a negative effect on the pore structure of mortar, thus affecting the permeability resistance of mortar. The test results showed that when the content of nanosilica sol is greater than 1%, the harmful pores in mortar will increase, which will damage the permeability resistance and strength performance of mortar. At the 28-day age of mortar, the D_RCM_ value of HBSAC cement mortar decreased by 7.7%, 13.2% and 16.7%, respectively when the content of nanosilica sol was 0, 1% and 2%. The D_RCM_ value of P·O 42.5 cement mortar decreased by 8.9%, 9.8% and 17% when the content of nanosilica sol was 0, 1% and 2%, respectively. This phenomenon may be due to the improvement of harmful pores of mortar by nanosilica sol particles.

## Figures and Tables

**Table 1:** Chemical composition of the raw materials by XRF (wt.%).

Chemical Composition	CaO	SiO_2_	Al_2_O_3_	Fe_2_O_3_	SO_3_	TiO_2_	MgO	Al_2_O_3_
HBSAC	51.5	13.8	15.3	1.5	14.2	0.7	2.1	6.4
P·O 42.5	52.7	19.9	6.4	2.8	2.6	0.4	/	/

**Table 2:** Mix proportion design of mortar.

Sample	Nanosilica Sol Content (%)	Water-Reducing Agent (%)	Fine Aggregate (g)	Cement (g)	Water (g)	Liquidity (HBSAC) (mm)	Liquidity (P·O 42.5) (mm)
1	0	0	1350	450	180	170	172
2	0.5	0.3	1350	450	180	170	170
3	1	0.6	1350	450	180	170	173
4	1.5	1.0	1350	450	180	170	171
5	2	1.4	1350	450	180	170	172

## Abbreviations

The following abbreviations are used in this manuscript:

HBSAC	High-belite sulphoaluminate cement
P·O 42.5	Ordinary Portland cement (42.5 grade)
CH	Ca(OH)_2_
AFt	3CaO·Al_2_O_3_·3CaSO_4_·32H_2_O
β-C_2_S	2CaO·SiO_2_
C_3_S	3CaO·SiO_2_
$C_{4}A_{3}\bar{S}$	3CaO·3Al_2_O_3_·CaSO_4_
